# Patient background and prognosis of chronic pulmonary aspergillosis in fibrosing interstitial lung disease

**DOI:** 10.1097/MD.0000000000029936

**Published:** 2022-08-12

**Authors:** Hideaki Yamakawa, Tomotaka Nishizawa, Hiroki Ohta, Yuta Tsukahara, Tomohiko Nakamura, Shintaro Sato, Rie Kawabe, Tomohiro Oba, Keiichi Akasaka, Masako Amano, Kazuyoshi Kuwano, Hiroki Sasaki, Hidekazu Matsushima

**Affiliations:** a Department of Respiratory Medicine, Saitama Red Cross Hospital, Chuo-ku, Saitama, Japan; b Department of Respiratory Medicine, Tokyo Jikei University Hospital, Tokyo, Japan; c Department of Radiology, Saitama Red Cross Hospital, Chuo-ku, Saitama, Japan.

**Keywords:** chronic pulmonary aspergillosis, interstitial lung disease, prognosis

## Abstract

Several previous reports have shown interstitial lung disease (ILD) to be a predictor of poor prognosis in patients with chronic pulmonary aspergillosis (CPA). However, there is a lack of clarity regarding patient background and the prognostic factors in CPA associated with ILD (CPA-ILD). Therefore, we assessed these points to obtain valuable information for clinical practice.

We retrospectively surveyed and collected data from 459 patients who had serum examination for anti-*Aspergillus* antibody. Of these patients, we extracted and investigated CPA-ILD patients.

We ultimately analyzed 32 CPA-ILD patients. Patient background factors more frequently showed the patients to be older (mean: 74.9 years), male (75.0%), and to have a smoking history (71.9%). Median survival time from the diagnosis of ILD was 76.0 months, whereas that from the diagnosis of CPA-ILD was 25.5 months. No significant differences in survival were found in regard to each ILD pattern and the presence of idiopathic pulmonary fibrosis. A higher level of C-reactive protein was a significant predictor of mortality by Cox regression analysis.

CPA complicating ILD is associated with poor prognosis. ILD patients with older age, male sex, and smoking history should be aware of the potential for the development of CPA in ILD. If such patients have elevated markers of inflammation, prompt induction of antifungal treatment may improve their prognosis. Clinicians should be aware of which complications of CPA may lead to a poor prognosis for any ILD not just those limited to idiopathic pulmonary fibrosis or usual interstitial pneumonia pattern.

## 1. Introduction

Chronic pulmonary aspergillosis (CPA) is an uncommon and problematic pulmonary disease.^[1]^ Although the treatment for CPA involves the use of long-term antifungal agents, the prognosis is generally poor.^[2,3]^ CPA occurs in patients without obvious immunosuppression but with underlying pulmonary disease, such as previous tuberculosis, nontuberculous mycobacterial pulmonary disease (p-NTM), pulmonary emphysema, and interstitial lung disease (ILD).^[2,4–6]^ Among these pulmonary disorders, previous reports showed that having ILD was a significant poor prognostic factor.^[4,7,8]^ Also in clinical practice, we often encounter CPA patients with ILD and then struggle with management of the CPA. Currently, there is a lack of clarity regarding patient background factors and prognosis specifically in ILD patients with CPA. Therefore, in the present study, we aimed to clarify these factors in ILD patients with CPA.

## 2. Materials and Methods

### 2.1. Study sample

This study was approved from the institutional review board of Saitama Red Cross Hospital (approval no. 21-V), which waived the need for patient approval or informed consent because the study involved a retrospective review of clinical records. We surveyed the medical records of 459 patients who had serum examination of anti-*Aspergillus* antibody between June 2014 and May 2021 at Saitama Red Cross Hospital, Japan. Among these patients, 88 had a positive serum anti-*Aspergillus* antibody result, of whom 4 patients with invasive aspergillosis (i.e., acute onset), 3 patients with allergic bronchopulmonary aspergillosis, and 2 patients with unknown disease were excluded. Thus, 79 patients were diagnosed as having CPA based on the criteria by Kimura et al,^[8]^ which was modified by the European Society for Clinical Microbiology and Infectious Diseases and the European Respiratory Society.^[1]^ We then excluded 3 patients with simple aspergilloma/*Aspergillus* nodule and 4 patients having no underlying respiratory disease. This resulted in 72 CPA patients with underlying respiratory disease comprising 32 patients with chronic ILD, 17 with pulmonary emphysema, 7 with previous tuberculosis, 6 with p-NTM, 4 with bronchiectasis, 3 with postoperative lung cancer, and 1 patient each with sarcoidosis, lung cancer, and pneumoconiosis, respectively. Finally, we extracted 32 patients who had CPA with chronic ILD and then collected data from these patients’ medical records that included characteristics, laboratory data, pulmonary function results, and chest computed tomography (CT) findings at the time of the CPA diagnosis. We collected baseline pulmonary function results within 6 months of the initial diagnosis of CPA at our hospital and extracted the other baseline clinical measurements within 3 months of the diagnosis. In addition, we analyzed CT patterns at the time of the ILD diagnosis. Survival was defined as the time from the date of either the CPA diagnosis or ILD diagnosis to the date of death or date of censoring.

### 2.2. Radiological analysis

Each subject’s radiological findings were reviewed by 2 expert pulmonologists (TN and HM) and 1 radiologist (HS). High-resolution CT (HRCT) patterns were classified as usual interstitial pneumonia (UIP), probable UIP, nonspecific interstitial pneumonia (NSIP), pleuroparenchymal fibroelastosis (PPFE), or unclassifiable.^[9,10]^ The unclassifiable classification was defined by a multiple HRCT pattern or difficulty in recognition due to the strong influence of smoking (i.e., smoking-related ILD).^[11–13]^ For combined pulmonary fibrosis with emphysema (CPFE), positive findings of emphysema were visually defined as the presence of an area of low attenuation indicating the lack of a distinct alveolar wall threshold of over 10%.^[14]^ Honeycomb pattern was defined as clustered cystic air spaces with well-defined walls and typically comparable diameters of 3 to 10 mm in subpleural and lower lobes.^[9]^ The diagnosis of idiopathic pulmonary fibrosis (IPF) was based on a multi-disciplinary discussion that considered HRCT pattern and disease behavior.^[9]^

### 2.3. Statistical methods

Categorical baseline characteristics are summarized by frequency and percentage, and continuous characteristic are reported as the mean ± SD. We investigated potential risk factors of mortality for each variable chosen for entry into univariate Cox regression analysis. Thereafter, the variables that achieved a modest level of statistical significance (*P* < .1 on univariate analysis) based on forward variable selection were assessed in a multivariate analysis. We considered *P* < .05 to indicate statistical significance. All data were analyzed with SPSS version 22.0 (IBM Japan, Tokyo, Japan).

## 3. Results

### 3.1. Overall patient characteristics

**Figure 1.**
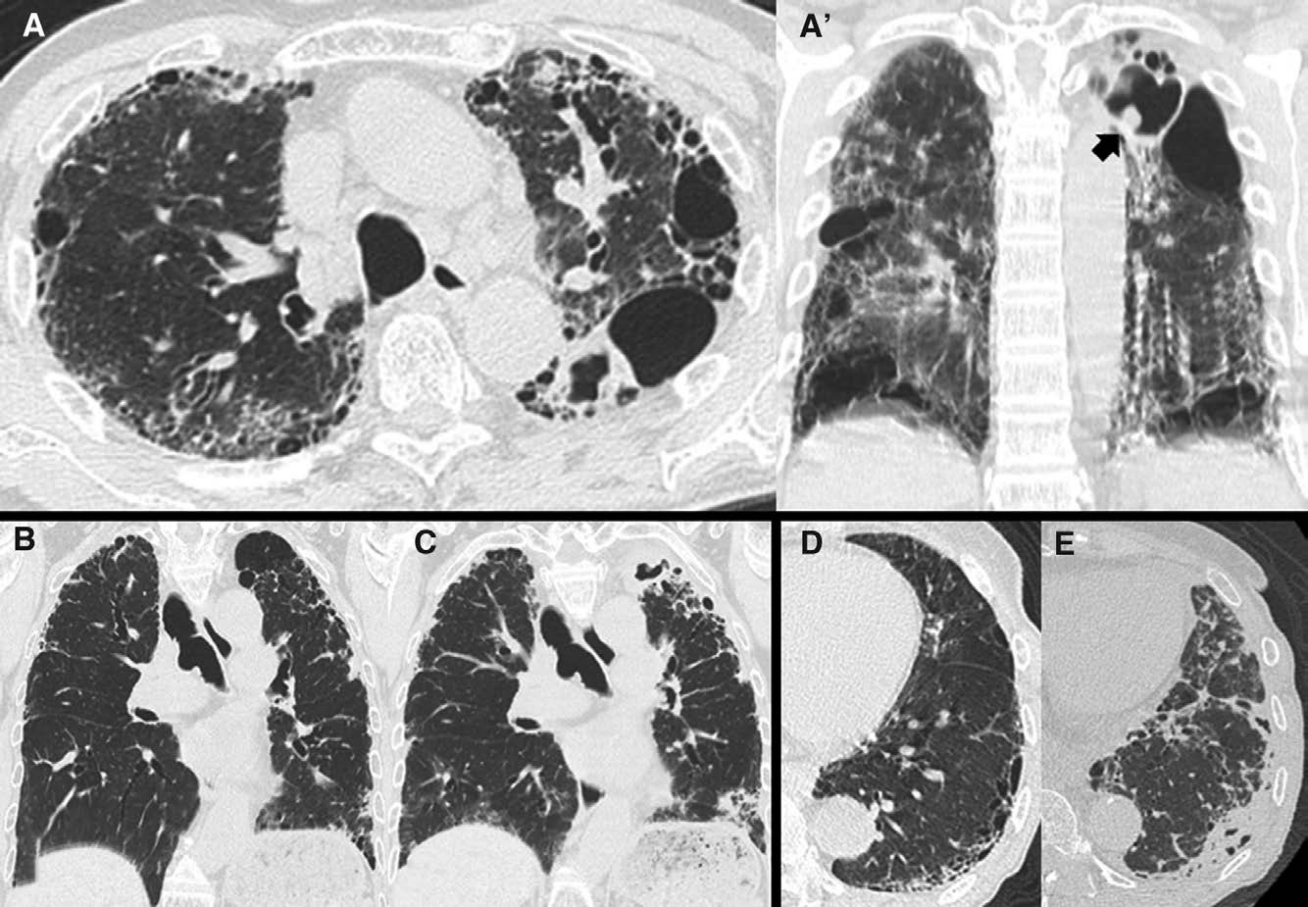
Chest CT images of typical cases of CPA. (A/A′) Chest CT scan of a 77-year-old man with emphysema with interstitial lung disease showed a fungus ball (black arrow) in the cavitary lesion with consolidation in the left upper lobe. (B/C) Chest CT scan of a 71-year-old man revealed fibrosing interstitial lung disease as pleuroparenchymal fibroelastosis in (B). In the same patient 4 months after initiation of corticosteroid therapy (C), pericystic infiltartion and pleural thickening in the left upper lobe of the CT image were apparent, and he was subsequenlty diagnosed as having CPA. (D/E) Chest CT scan of a 72-year-old woman revealed reticulation with traction bronchiectasis in the subpleural lung as probable UIP pattern (D). In the same patient 2 years and 2 months later (E), localized consolidation was apparent in the subpleural fibrotic lesion, and she was diagnosed as having CPA. CPA = chronic pulmonary aspergillosis, CT = computed tomography.

The study cohort included 32 CPA patients with chronic ILD. Among the disease types of CPA in these patients, only 1 patient had chronic cavitary pulmonary aspergillosis overlapping with subacute invasive aspergillosis; most patients had chronic cavitary pulmonary aspergillosis or chronic fibrosing pulmonary aspergillosis (a typical example is shown Fig. 1A/A′). As shown in Table 1, the mean age of the patients with CPA-associated ILD was 74.9 years, 75.0% were male, and the proportion of patients who smoked was 71.9%. Mean serum values were 3.5 mg/dL for albumin, 718.6 U/mL for KL-6, and 1.9 mg/dL for C-reactive protein (CRP). Eleven patients (34.4%) were diagnosed as having IPF and 14 (43.8%) as having CPFE. Ten patients (31.3%) were receiving home oxygen therapy at the time of CPA diagnosis. Comorbidities at the time of CPA diagnosis were rheumatoid arthritis in 4 patients (12.5%), diabetes mellitus in 7 patients (21.9%), malignancy in 2 patients (6.3%), and p-NTM in 3 patients (9.4%). Pulmonary function testing showed a mean % forced vital capacity of 81.8% and mean % diffusing capacity of the lung for carbon monoxide of 60.6%. The HRCT patterns at the time of ILD diagnosis were UIP in 13 patients (40.6%), probable UIP in 2 patients (6.3%), NSIP in 1 patient (3.1%), PPFE in 4 patients (12.5%), and unclassifiable in 12 patients (37.5%). The unclassifiable HRCT patterns included a multiple mixed pattern (N = 3 [9.4%]) and smoking-related ILD (N = 9 [28.1%]). The most frequent site of *Aspergillus* infection was the right upper lobe (62.5%). As treatment for ILD at the time of CPA diagnosis, 9 patients (28.1%) received corticosteroid, 6 patients (18.8%) received immunosuppressive agents (i.e., cyclosporin, tacrolimus, methotrexate, and abatacept), and 7 patients (21.9%) received antifibrotic agents (i.e., pirfenidone or nintedanib). Typical examples of disease courses are shown in Figure 1B, C, D, E. Of the 32 patients with CPA associated with ILD, 17 patients (53.1%) received antifungal treatment regardless of the duration of treatment.

**Table 1:** Patient characteristics (N = 32).

Male, N (%)	24 (75.0%)
Age, mean ± SD	74.9 ± 6.3
Current or exsmoker, N (%)	23 (71.9%)
Body mass index (kg/m^2^), mean ± SD	20.6 ± 3.5
Albumin (g/dL), mean ± SD	3.5 ± 0.6
KL-6 (U/mL), mean ± SD	718.6 ± 361.5
CRP (mg/dL), mean ± SD	1.9 ± 2.6
Receive HOT, N (%)	10 (31.3%)
IPF, N (%)	11 (34.4%)
HRCT pattern at the time of ILD diagnosis	
UIP	13 (40.6%)
Probable UIP	2 (6.3%)
NSIP	1 (3.1%)
PPFE	4 (12.5%)
Unclassifiable (mixed pattern/smoking related)	3 (9.4%)/9 (28.1%)
CPFE, N (%)	14 (43.8%)
%FVC, mean ± SD (available N = 21)	81.8 ± 18.8
%DL_CO_, mean ± SD (available N = 20)	60.6 ± 22.3
Location of CPA	
Right upper lobe	14 (43.8%)
Right lower lobe	4 (12.5%)
Left upper lobe	4 (12.5%)
Left lower lobe	1 (3.1%)
Left upper and lower lobes	1 (3.1%)
Right upper and left upper lobe	6 (18.8%)
Right lower and left lower lobes	2 (6.3%)
Treatment for ILD at the time of CPA diagnosis	
Corticosteroid	9 (28.1%)
Immunosuppressive agents	6 (18.8%)
Antifibrotic agents	7 (21.9%)
Treatment for CPA	
Surgery, N (%)	1 (3.1%)
Azole antifungal agents	12 (37.5%)
Echinocandin antifungal agents	8 (25.0%)
Liposomal amphotericin B	1 (3.1%)
Deaths (during follow-up), N (%)	20 (62.5%)

### 3.2. Survival

**Figure 2.**
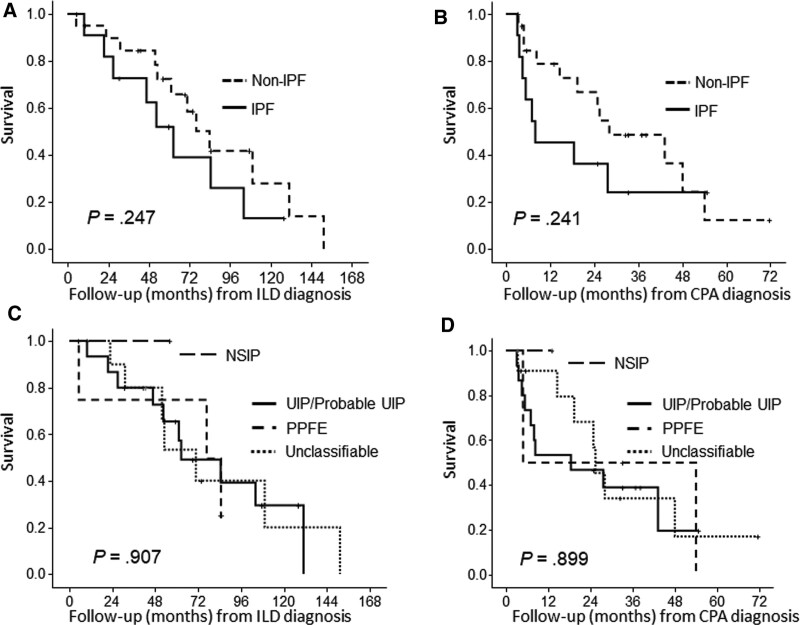
Kaplan-Meier survival curves of all-cause mortality at the diagnosis of ILD and following the diagnosis of CPA. (A) IPF patients showed no significant difference in survival compared with the non-IPF patients during the follow-up period from ILD diagnosis (*P* = .247; median survival time: IPF, 62.5 months vs. non-IPF, 83.9 months). (B) There was also no significant difference in survival after the CPA diagnosis (*P* = .241; median survival time: IPF, 8.0 months vs. non-IPF, 28.1 months). In regard to the HRCT pattern at the time of the ILD diagnosis, there was no significant difference in survival of patients with each pattern from the date of either (C) the ILD diagnosis (*P* = .907; median survival time: UIP or probable UIP, 62.6 months, PPFE, 76.0 months, and unclassifiable, 70.7 months) or (D) that of the CPA diagnosis (*P* = .899; median survival time: UIP or probable UIP, 18.4 months, PPFE, 4.8 months, and unclassifiable, 25.5 months). CPA = chronic pulmonary aspergillosis, HRCT = high-resolution CT, ILD = interstitial lung disease, IPF = idiopathic pulmonary fibrosis, PPFE = pleuroparenchymal fibroelastosis.

The patients were observed over a median follow-up period of 18.9 (range: 3.1–54.0) months from the CPA diagnosis and 57.6 (range: 4.8–151.3) months from the ILD diagnosis. During the follow-up period, 20 patients died (62.5%). Among these patients, the direct cause of death as indicated in the medical record involved acute exacerbation of ILD (N = 4), chronic progression of ILD (N = 8), lung cancer (N = 2), respiratory tract infection (N = 4) including CPA itself (N = 1), gastric cancer (N = 1), and pneumothorax (N = 1). The respective median and mean overall survival times from the ILD diagnosis were 76.0 months and 78.9 months, and those from the CPA diagnosis were 25.5 months and 30.8 months. Kaplan-Meier survival curves and log-rank test showed no significant differences in survival between the IPF patients and non-IPF patients from the viewpoints of the follow-up period from the ILD diagnosis (Fig. 2A, *P* = .247; median survival time: IPF, 62.5 months vs. non-IPF, 83.9 months) and that from the CPA diagnosis (Fig. 2B, *P* = .241; median survival time: IPF, 8.0 months vs. non-IPF, 28.1 months). In regard to the HRCT pattern at the time of the ILD diagnosis, there was no significant difference in the survival of patients with each pattern from the date of either the ILD diagnosis (Fig. 2C, *P* = .907) or CPA diagnosis (Fig. 2D, *P* = .899). Also, in the patients in whom CPFE was present, there was no significant difference in survival from the date of either the ILD diagnosis (*P* = .859) or CPA diagnosis (*P* = .753). CRP was a significant predictor of mortality by univariate and multivariate Cox regression analyses (Table 2). We could not determine the optimal cutoff value for CRP that would represent increased poor prognosis following the diagnosis of CPA associated with ILD for any follow-up periods examined (e.g., 6 months, 1 or 3 years, and overall follow-up).

**Table 2:** Analysis of predictors of mortality.

	Univariate Cox regression			Multivariate Cox regression		
	HR	95% CI	*P*	HR	95% CI	*P*
Male	0.724	0.257–2.038	.541	–		
Age	0.995	0.930–1.063	.873	–		
Current/ex-smoker	0.841	0.311–2.274	.733	–		
Body mass index	1.011	0.881–1.159	.879	–		
Albumin	0.619	0.288–1.328	.218	–		
KL-6	1.000	0.999–1.001	.768	–		
CRP	1.328	1.081–1.632	**.007**	1.328	1.081–1.632	**.007**
Receive HOT	2.279	0.830–6.259	.110	–		
IPF (vs. non-IPF)	1.705	0.692–4.199	.246	–		
HRCT pattern				–		
UIP or probable UIP	1.000	ref				
PPFE	0.906	0.245–3.356	.883			
NSIP	n.c.					
Unclassifiable	0.816	0.309–2.152	.680			
CPFE	0.867	0.357–2.104	.753	–		
%FVC	0.967	0.926–1.011	.144	–		
%DL_CO_	0.985	0.958–1.013	.302	–		
Treatment for ILD						
Corticosteroid	1.567	0.586–4.188	.371	–		
Antifibrotic agents	2.361	0.862–6.465	.095	n.e.		

## 4. Discussion

*Aspergillus* infection is usually seen in those with underlying respiratory disease. However, the report of its association with chronic ILD is rare.^[7]^ Therefore, we focused on the correlations between CPA and ILD in the present study and then investigated both the patient characteristics and the prognosis or mortality risk of CPA in patients with chronic ILD.

In general, in reports of the background of patients with CPA, older age (median: 68.5–73.4 years), male sex (73.9–80.4%), and a smoking history (69.7–71.1%) were predominant, as in a present study.^[4,7,8,15]^ These characteristics were similar in IPF cohorts.^[16,17]^ When limited to CPA associated with ILD, the proportion of patients with IPF was 60.0% as reported by Kurosaki et al^[7]^ and 34.4% in the present study. Therefore, any ILD patients having these particular factors (i.e., older age, male sex, and a smoking history) should be aware of the potential complication of CPA not only in IPF but also in any ILD.

In addition, the site of *Aspergillus* infection was predominantly in the upper lobes as previously reported.^[7,18]^ In the present study, UIP pattern with honeycombing (40.6%) and smoking-related ILD (28.1%) accounted for the majority of cases of *Aspergillus* infection. The frequency of home oxygen therapy use was high (73.3%) as reported by Kurosaki et al^[7]^ and 31.3% in the present study, indicating that the complication of CPA can occur easily in patients with a progressive phase of ILD. In other words, wide-spread cystic lesions and emphysema as destructive changes in lung tissue can be colonized by *Aspergillus*. Although we could not clearly determine why the lesions of CPA were located predominantly in the upper lobes, 1 reason might be that chronic lung infection causes the loss of alveoli (e.g., due to cystic lesions and emphysema) and elastic recoil in the chest wall that results in air trapping, which induces a tendency for lower compliance and movement of the thoracic cage primarily in the upper lobes that may influence the development of CPA.^[19]^

Previously reported negative prognostic factors in CPA were older age, male sex, lower body mass index, lower albumin, corticosteroid use, and higher CRP.^[4,8]^ The present study also showed higher CRP to be a negative prognostic factor in CPA associated with ILD. The response to antifungal therapy in CPA is generally low, and the optimal duration of therapy is unknown.^[1]^ This background and the progression of ILD itself might be influencing factors that resulted in more than 40% of patients not receiving antifungal treatment in the present study. However, if the patients with CPA associated with ILD have elevated markers of inflammation such as CRP, prompt induction of antifungal treatment may improve their prognosis if higher CRP is responsible for CPA. Points to be careful that CRP is a nonspecific inflammatory marker and many conditions can cause its rise, therefore, clinicians should think the reason in each patients having higher CRP. In addition, corticosteroid use was reported to be 1 of the major risk factors for CPA, posing a significant risk of progression or dissemination.^[1]^ Corticosteroid have often been used in non-IPF patients, and in fact, it was used in just under 30% of the patients in the present study. Clinicians need to pay adequate attention to this, even though this factor was not found to be a significant prognostic factor in the present study.

Interestingly, an IPF or UIP pattern on HRCT was not a significant factor affecting prognosis, even though these patterns have been commonly associated with poor prognosis.^[9,11,20,21]^ Although the small sample size of the present study might strongly influence the results, the median survival time from the CPA diagnosis was poor (25.5 months) in the study patients. Taken together, CPA itself may lead to a poor prognosis for any ILD patient regardless of the presence or absence of IPF or UIP.

Our study has several limitations. First, it is a retrospective, single-institution study, which can introduce referral bias and limit the ability to generalize our findings. Second, the CPA diagnosis included patients positive for serum anti-*Aspergillus* antibody. Although the presence of anti-*Aspergillus* antibody can differentiate between infected and colonized patients with a relatively high positive predictive value, some patients with a CPA diagnosis might have colonization or false-positive results for anti-*Aspergillus* antibody.^[15]^ Third, we could not quantitatively assess the HRCT patterns, and this will be an issue for further investigation. Fourth, CRP, being the single independent predictor factor for poor prognosis might be not a satisfactory finding. This is because small number of the enrolled patients might be the contributing factor for such a finding and could be a major limitation.

## 5. Conclusions

As previous studies, the present study also revealed that many of the patients with CPA associated with ILD had a background of older age, male sex, and a smoking history. Additionally, higher CRP was a negative prognostic factor among the patients with CPA associated with ILD. In patients with any type of ILD, regardless of whether it is IPF, and a HRCT UIP pattern, a higher CRP can place them at higher risk of having a poor prognosis after the CPA diagnosis. As the complication of CPA may lead to a poor prognosis for any ILD, clinicians should be aware of the importance of a prompt, adequate diagnosis and having a decision-making strategy for the treatment of CPA associated with ILD.

## Acknowledgments

We sincerely thank Yosuke Sasaki of Satista Co., Ltd., for his advice on statistical analysis. The authors would like to thank Rise Japan LLC for the professional English language review.

## Author contributions

**Investigation:** Hideaki Yamakawa, Tomotaka Nishizawa

**Methodology:** Tomotaka Nishizawa

**Project administration:** Hideaki Yamakawa, Hiroki Ohta, Masako Amamo

**Software:** Hideaki Yamakawa

**Supervision**: Keiichi Akasaka, Masako Amano, Hiroki Sasaki, Kazuyoshi Kuwano

**Validation:** Yuta Tsukahara, Tomohiko Nakamura, Tomohiro Oba

**Visualization:** Shintaro Sato, Rie Kawabe

**Writing—original draft:** Hideaki Yamakawa, Hidekazu Matsushima
